# Transcriptome Analysis of the Hippocampal CA1 Pyramidal Cell Region after Kainic Acid-Induced Status Epilepticus in Juvenile Rats

**DOI:** 10.1371/journal.pone.0010733

**Published:** 2010-05-20

**Authors:** Hanna B. Laurén, Francisco R. Lopez-Picon, Annika M. Brandt, Clarissa J. Rios-Rojas, Irma E. Holopainen

**Affiliations:** 1 Department of Pharmacology, Drug Development, and Therapeutics, Institute of Biomedicine, University of Turku, Turku, Finland; 2 MediCity Research Laboratory, Turku, Finland; 3 Turku Centre for Biotechnology, University of Turku and Åbo Akademi University, Turku, Finland; University of North Dakota, United States of America

## Abstract

Molecular mechanisms involved in epileptogenesis in the developing brain remain poorly understood. The gene array approach could reveal some of the factors involved by allowing the identification of a broad scale of genes altered by seizures. In this study we used microarray analysis to reveal the gene expression profile of the laser microdissected hippocampal CA1 subregion one week after kainic acid (KA)-induced status epilepticus (SE) in 21-day-old rats, which are developmentally roughly comparable to juvenile children. The gene expression analysis with the Chipster software generated a total of 1592 differently expressed genes in the CA1 subregion of KA-treated rats compared to control rats. The KEGG database revealed that the identified genes were involved in pathways such as oxidative phosporylation (26 genes changed), and long-term potentiation (LTP; 18 genes changed). Also genes involved in Ca^2+^ homeostasis, gliosis, inflammation, and GABAergic transmission were altered. To validate the microarray results we further examined the protein expression for a subset of selected genes, glial fibrillary protein (GFAP), apolipoprotein E (apo E), cannabinoid type 1 receptor (CB1), Purkinje cell protein 4 (PEP-19), and interleukin 8 receptor (CXCR1), with immunohistochemistry, which confirmed the transcriptome results. Our results showed that SE resulted in no obvious CA1 neuronal loss, and alterations in the expression pattern of several genes during the early epileptogenic phase were comparable to previous gene expression studies of the adult hippocampus of both experimental epileptic animals and patients with temporal lobe epilepsy (TLE). However, some changes seem to occur after SE specifically in the juvenile rat hippocampus. Insight of the SE-induced alterations in gene expression and their related pathways could give us hints for the development of new target-specific antiepileptic drugs that interfere with the progression of the disease in the juvenile age group.

## Introduction

Epilepsy, one of the most common neurological disorders affecting up to 1% of the population, is caused by a number of both unknown and known factors such as trauma, hypoxia, postnatal insults, and status epilepticus (SE) [1], [2]. In experimental animal models, SE can be induced by different chemoconvulsants, e.g. kainic acid (KA), which induces region-specific neuropathological changes in the hippocampus comparable to those of patients with chronic temporal lobe epilepsy (TLE) [3], [4], [5]. In addition to region-specificity, earlier studies indicate that the extent and localization of KA-induced hippocampal damage is age-specific, the immature rats (<21-day old) having minor or even no obvious damage [6], [7], [8], while degeneration of CA1 and CA3 pyramidal neurons and hilar interneurons have frequently been documented in adult rats [3], [6], [9].

The acute seizure-induced excitotoxic insult is known to initiate a process of changes defined as epileptogenesis, which finally leads to spontaneous seizures, i.e. epilepsy in the majority of adult rats [1], [10], [11]. In immature rats, the appearance of spontaneous seizures and other long-term consequences seems to be less severe [6], [10], [12]. In spite of numerous studies focusing on epileptogensis both in adult and immature brain, its cellular and molecular mechanisms have remained largely undiscovered. In search for factors involved in epileptogenesis in the hippocampus, the gene expression approach using microarrays has recently been successfully applied. The results of these studies suggest that the expression of a number of genes is altered in the adult rat hippocampus after SE induced by KA [13], [14], pentylenetetrazol [15], and electrical stimulation [16], [17]. The results of more detailed gene microarray studies carried out in specimen microdissected from the selected hippocampal sub-regions have revealed that the alterations are not uniform, but region-specifically distributed in the hippocampus [18], [19], [20], [21], [22]. It can be assumed that the targeted gene array approach reveals more exactly the region-specific pathways activated in the course of epileptogenesis. Furthermore, as seizure-induced pathology seems to be age-specific, there could also be age-specific differences in the pathways altered by seizures, which could elucidate in more detail the postulated differences in epileptogenesis between the immature and mature brain. However, to our knowledge, there is only one earlier microarray study focusing on gene expression in normal immature rats (P3) [21], and no earlier studies after experimental SE either in immature or juvenile animals.

In our current study, we specifically focused on juvenile, 21-day-old (P21) rats, in which KA-induced SE leads to selective damage of hippocampal CA1 pyramidal neurons while saving neurons of the other sub-regions [7], [23]. We searched for alterations in the gene expression pattern during the early epileptogenetic phase, i.e. one week after SE, and compared the results with those of age-matched control rats. To detect specifically changes in the CA1 pyramidal neurons, we used the laser-capture microdissection technique that allows the precise isolation of the region of interest. The transcriptome was analyzed by using gene expression microarrays which generated differentially expressed genes, and the results of certain, selected genes of interest were confirmed at the protein level using immunohistochemistry.

## Materials and Methods

### Kainate treatment of the rats

P21 Sprague–Dawley male rats were chosen for these experiments (n = 4, in both the control and KA-treated groups). A single dose of KA (Tocris Cookson Ltd., Avomouth, UK) (7 mg/kg) was injected intraperitoneally to the rats, which were thereafter carefully followed up to detect signs of seizures, as earlier described in detail [23]. Within 15 min after the injection, rats first showed deep breathing and increased salivation followed by scratching with further progression to rearing and generalized tonic/clonic seizures within 50–60 min, which lasted for 2–3 h. After a follow-up for further 2 h after the cessation of behavioral seizures, rats were taken back to their cages, and sacrificed 7 days after the KA injection. Control rats received the same volume of 0.9% NaCl as those of KA-treated, but were otherwise treated as the KA-injected rats described above. About 80% of KA-treated animals developed SE, and only animals which experienced SE were included in this study. All animal procedures were conducted in accordance with the guidelines of the European Community Council Directives 86/609/EEC, and had the approval of the Office of the Regional Government of Western Finland. Using our protocol the mortality of rats was zero. All efforts were made to minimize the pain, discomfort, and number of experimental animals.

### Tissue collection

**Figure 1:**
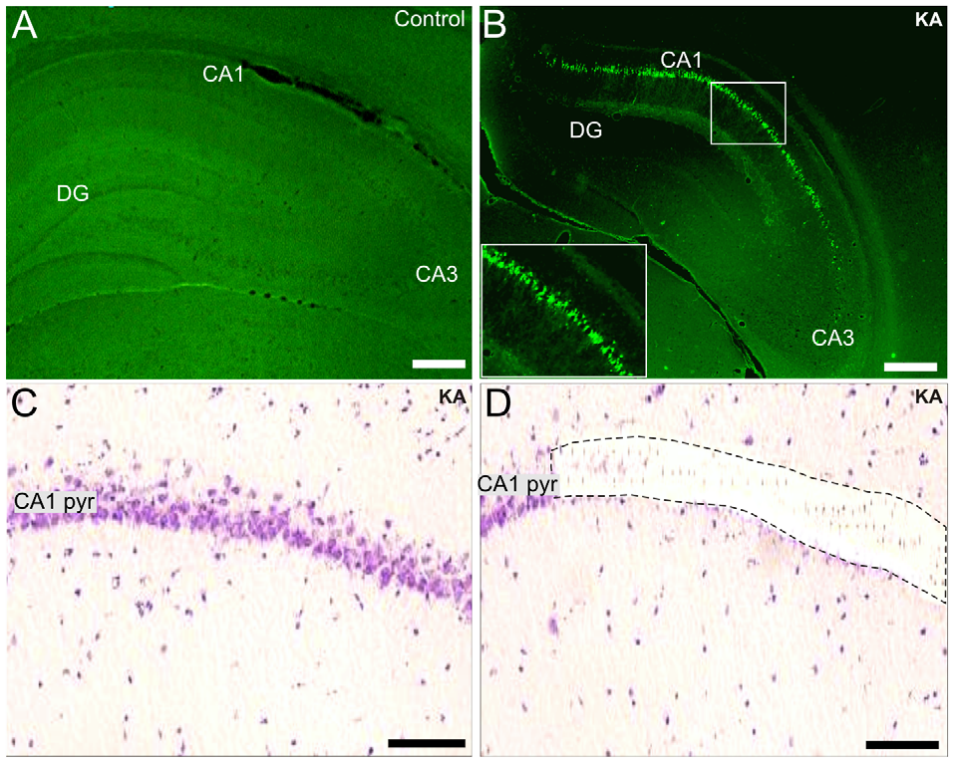
Neuronal CA1 pyramidal cell damage and the region-specific laser microdisecction one week after SE. Fluoro-Jade B staining of a representative control (A) and KA-treated rat (B) one week after SE. Fig.1B shows positively stained CA1 pyramidal neurons (see also the inset in B). Figs.1C and D show hematoxylin-eosin staining of the CA1 pyramidal layer of a KA-treated rat before and after laser microdissection, respectively. Scale bars: A and B: 200 µm; C and D: 75 µm. Abbreviations: CA1 pyr, stratum pyramidale of CA1; DG, dentate gyrus; KA, kainic acid.

After decapitation, brains where dissected into halves. One half of the brain was rapidly frozen in liquid nitrogen for the microarray study, and the other half was submerged in 4% paraformadehyde (PFA) in phosphate-buffered saline (PBS, pH 7.4) for immunocytochemistry and Fluoro-Jade B (FJB) staining. For the transcriptome studies, the liquid nitrogen-frozen brains were cut into 40 µm coronal slices onto PALM membrane slides (PALM Microlaser Technologies GmbH, Germany), and stored at −80°C. To visualize the various hippocampal subregions, all slices prepared from the hippocampi of control and KA-treated rats were stained with hematoxylin-eosin, and the hippocampal pyramidal CA1 cell layer of the control and KA-treated rats was cut using the laser capture micro-dissection (LCM) technology, and catapulted on PALM adhesive caps (see Fig. 1D). Eight consecutive sections obtained from each animal were used to maximize the yield and quality of the extracted total RNA and to circumvent the need of additional PCR-amplification steps for the subsequent microarray labelling of the RNA-samples.

### RNA isolation and assessment of the RNA quality

The total RNA of the LCM samples was isolated using the EPICENTRE Array Pure™ Nano – scale RNA Purification Kit (Madison, USA). The RNA solution was treated with the DNase and RNase inhibitor (ScriptGuard™, included in the Array Pure™ Nano Kit), and the quality and approximate quantity of the resulting RNA were determined spectrofotometrically using Nanodrop-1000 (Thermo Fischer Scientific, Wilmington, USA), and a capillary electrophoresis system (Experion, Bio-Rad).

### Microarray analysis

Total RNA (100 ng), isolated from LCM-samples that were dissected from each individual experimental animal separately as described above, was amplified, labeled, and hybridized according to the manufacturers' protocols (Illumina, San Diego, CA, USA). In brief, biotin-labeled cRNA was prepared by a linear amplification method using the Illumina® TotalPrep™ RNA Amplification Kit. After the first and second strand synthesis, the cDNA served as the template for an *in vitro* transcription reaction to produce the biotinylated, amplified target cRNA (750 ng) used for the Illumina RatRef-12 Expression BeadChip arrays with each sample hybridized on separate arrays. After hybridization and washing the arrays were scanned and read by the Illumina Bead Array Reader, and the data was extracted by using the Illumina Bead Studio (version 3). All microarray data was deposited in ArrayExpress with the accession number E-TABM-881, according to the guidelines of MIAME by the European Bioinformatics Institute (www.ebi.ac.uk/microrarray_as/).

### Microarray data analysis

Microarray data analysis (Chipster software v1.1.0, CSC, Finland) was applied to identify those genes that had statistically significant differences in their expression between the groups. The outliers were excluded based on their expression intensity within a group, and those samples did not affect the outcome of the further analysis. In order to distinguish the two groups under assessment, phenodata was created, and the normalization of genes was made by the quantile normalization method (which makes the expression value to follow the same distribution on all chips), with no production of flags on the RatRef-12 chip type, beadstudio version 3, and by using “Probe ID” as identifier. In order to discard unreliable and uninformative data points, the filtering process was carried out, and the group of genes that have a corrected p-value smaller than the cut-off p-value (0.05) passed the statistical “two group test”. The filtering was performed by using the empirical Bayes method with Benjamin and Hochberg *post hoc* test, and the contrasts were created by using the Limma R/Bioconductor (www.bioconductor.org). Finally, the data was clustered by using the non-hierarchical K-means clustering for genes, which identifies *k* points that function as cluster centers. Each data point was then assigned to one of these centers in a way that minimizes the sum of the distances between all points and their centers [24]. The pathway analysis was performed by ‘Gene set test’, which analyzes the statistical significance of a set of genes simultaneously ranked by p-value and generates the KEGG categories (Chipster manual). The KEGG pathway maps and the gene ontology classification were obtained using the DAVID Bioinformatics Resources from the National Institute of Allergy and Infectious Diseases (NIAID), NIH (http://david.abcc.ncifcrf.gov/).

### Immunohistochemistry

Immunohistochemistry was carried out to verify the gene array results of certain, selected genes of interest. For that, brains of the KA-injected and their respective saline controls were used for the immunohistochemical study, which was carried out as previously described in detail [25]. After fixation in 4% PFA in PBS (pH 7.4), brains were cryoprotected in 30% sucrose in PBS at +4°C, frozen, and thereafter kept at −80°C until used. For the immunostaining, brains were cryosectioned in 30 µm slices, collected in PBS (pH 7.4), and processed in a free-floating system. Slices were first incubated in the blocking solution (BS) containing 2% bovine serum albumin, 2% goat serum, and 0.1% Triton X-100 in PBS (pH 7.4) for 1 h at room temperature, and thereafter with the primary antibodies for 24–48 h at +4°C in BS at the following dilutions: glial fibrillary acid (GFAP; 1:3000, Sigma, St. Louis, USA), apolipoprotein E (apo E; 1∶1000, Abcam, Cambridge, UK), cannabinoid type 1 receptor (CB1; 1∶1000, Abcam), Purkinje cell protein 4 (PEP-19; 1∶500, Santa Cruz Biotechnology, Santa Cruz, CA, USA), and interleukin 8 receptor (CXCR1; 1∶1000, Abcam). After washing in PBS, slices were incubated with the biotin-conjugated secondary antibody (1∶4000) in BS, rinsed with PBS, and incubated with the avidin-peroxidase conjugate (Vectastain ABC Kit, Vector Laboratories, Burlingame, CA, USA) in BS for 1 h at room temperature. The staining was detected using 3,3-diaminobenzidinetetrahydrochloride (Sigma) as a chromogen, and further processed as earlier described [25]. Alternatively, Alexa 568 (1∶2000, Invitrogen) was used as a secondary antibody. In each experiment, KA-treated and control brains of the same age were processed simultaneously, and two to three slices from each brain, in which the primary antibody was omitted but which were otherwise treated as indicated above, served as negative control. Preparations were examined with a Leica DM R microscope (Heerbrugg, Switzerland) under the bright field or fluorescence optics.

### Fluoro-Jade B staining

FJB, an anionic fluorescein with excitation peaks at 362 and 390 nm and the emission peak at 550 nm, is a marker of neuronal degeneration regardless of the nature of the injury [26]. FJB staining was carried out in brain slices prepared from 4% PFA-fixed brains 7 days after the KA injection. Brain slices (20 µm thick) were cut and mounted on gelatin-coated glasses. The FJB staining was carried out as previously described in detail [8] with minor modifications. Briefly, brain slices were rehydrated with alcohol series, transferred for 2–5 min to 0.06% potassium permanganate (KMnO_4_), washed with distilled water, and transferred to 0.001% FJB solution for 30 min. After that, slides were washed, dried, cleared in xylene, coverslipped with mounting medium, and examined with the Leica DM R microscope under fluorescence filters.

### Hematoxylin-Eosin staining

The hematoxylin-eosin staining was performed using basic histological staining protocols. Briefly, brain slices (40 µm slices on PALM membrane slides) were stained with Mayer's Hematoxylin, dehydrated in ethanol series, and cleared in xylene before using the LCM.

## Results

### Selective neuronal damage after SE in the CA1 region

The extent of regional hippocampal neuronal damage after KA-induced SE was assessed using FJB staining. Fig. 1A shows a representative image of the hippocampus of a P21 saline-injected control rat, in which no positively FJB-stained neurons were detected. In contrast, numerous FJB-stained pyramidal neurons appeared exclusively in the CA1 region one week after KA-induced SE (Fig. 1B). The hematoxylin-eosin staining (Fig. 1C) accurately indicated the CA1 pyramidal cell layer, which did not show any obvious neuronal loss. This region was microdissected with laser (Fig. 1D), and further processed to extract the total RNA.

### Microarray data analysis profile

**Figure 2:**
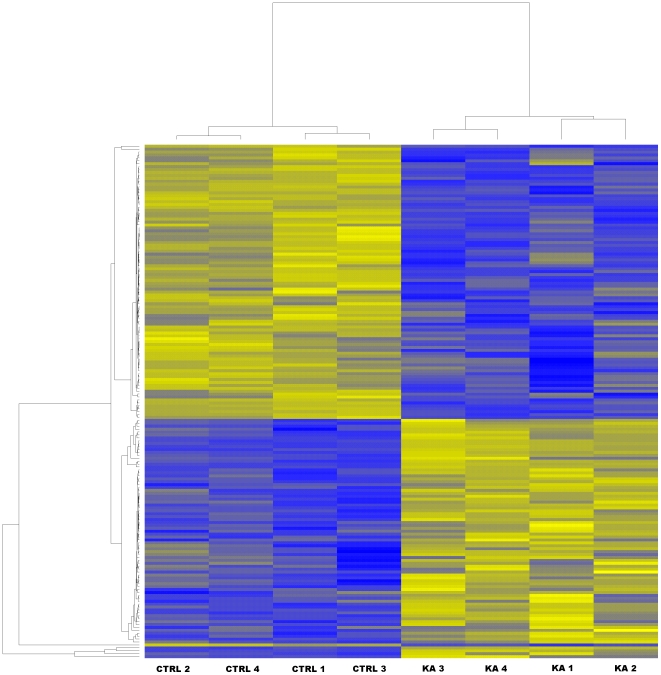
Heatmap of the samples assayed by microarrays. The figure illustrates the highest contrasts between the samples that were clustered by similarity in the gene expression levels. The control samples were clearly distinguished as a coherent group separate from the samples of KA-treated rats. Controls showed up-regulation of a specific set of genes (as indicated by yellow) in contrast to the samples of KA-treated rats presenting down-regulated levels of the same genes (as indicated by blue), while a distinct set of genes were down-regulated in the control samples, and up-regulated in the samples of KA-treated rats. The clustering of the genes (gene names omitted) is shown in the left vertical panel, and the clustering of the samples is indicated in the top horizontal panel of the figure. Abbreviations: CTRL, control rat; KA, kainic acid-treated rat.

**Figure 3:**
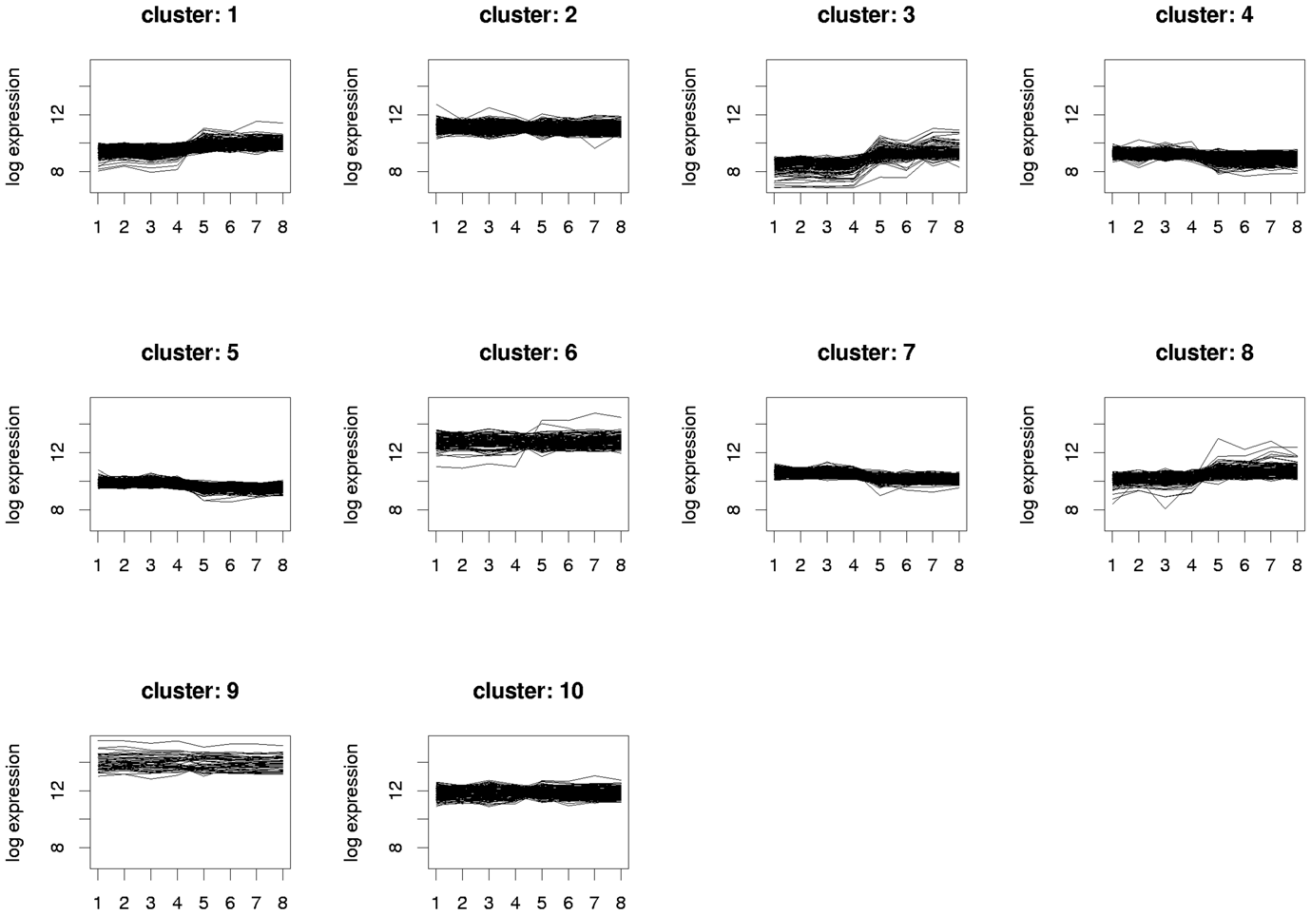
K-means clustering of the genes by their expression pattern. The K-means clustering method is visualized with the 10 distinct clusters as plotted by the log of the gene expression level of all the genes analyzed by the microrarrays. Our interest was to reveal the major differences between the KA-treated and control rats. In the figure the samples of the control and KA-treated rats are indicted by the numbers 1–4 and 5–8, respectively, in the x-axis. Abbreviation: KA, kainic acid.

Transcriptional responses of the CA1 region with the subsequent Illumina microarray analysis returned a total of 1592 genes that were statistically differentially expressed in KA-treated as compared to the age-matched control animals with the filter cut-off set at the 2-fold difference in the gene expression level with the p-value <0.05. The contrasts in the gene expression levels that were revealed by the data analysis between the KA-treated and control animals were visualized by a heatmap (Fig. 2), in which the yellow colour indicated up-regulation, and the blue colour down-regulation in the contrasted samples. The heatmap of the samples showed that the contrasting algorithm divided the samples in two distinct groups with the replicates of the control samples in one group (n = 4), and the samples from KA-treated rats in the other group (n = 4) (Fig. 2). The low variation between the samples from KA-treated rats may be due to the fact that all the treated animals included in this study experienced SE, and showed concomitant neurodegeneration in the CA1 pyramidal cell layer. This may have contributed to the high correlations between the biological samples within the groups (Fig. 2), that in turn resulted in a relatively high number of genes with a statistically significant difference in their expression. Further data mining of the transcriptome analysis included K-nearest neighbour classification. Using the K-means method the genes were classified in 10 different clusters representing the functionally distinct gene expression patterns that visualize the full extraction of the biologically significant phenomena in the data (Fig. 3).

### Genes related to pathways

**Figure 4:**
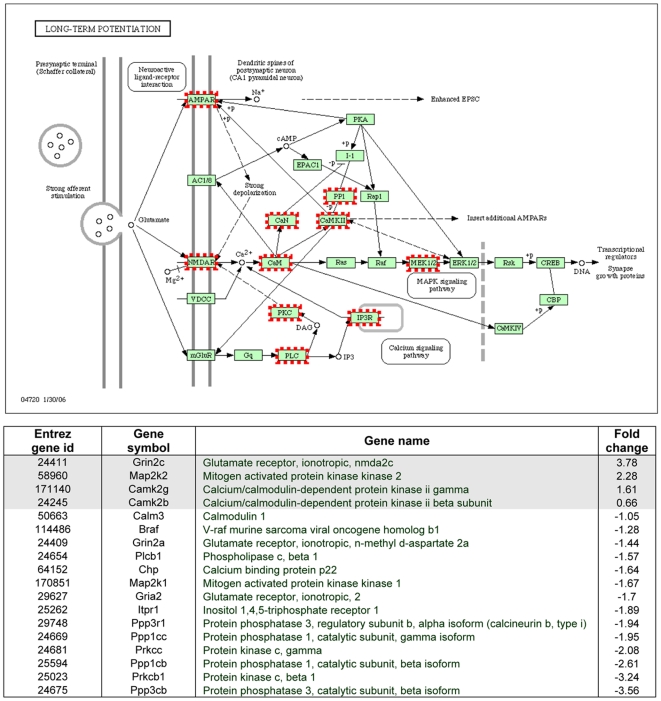
The KEGG-derived long-term potentiation pathway. The concerted action of glutamate on NMDA and AMPA receptors in CA1 pyramidal neurons is illustrated in this pathway, as well as the activation of different subpathways. The genes activated on the microarray are encircled. The table below shows the entire list of up- or down-regulated genes related to LTP on the microarray. Abbreviations: AMPA, α-amino-3-hydroxy-5-methyl-4-isoxazolepropionic acid; NMDA, N-methyl-D-aspartate.

The subsequent KEGG-test for the probe set over-representation analysis revealed the 15 significantly (p<0.05) changed KEGG-pathways in response to KA-treatment (see Materials and Methods and Table 1). Some of the most pronouncedly affected pathways were involved in oxidative phosphorylation (26 genes, of which 19 down-regulated; Fig. S1), ribosomal pathway (19 genes, of which 18 up-regulated; Fig. S2), long-term potentiation (LTP; 18 genes, of which 14 down-regulated; Fig. 4), and vascular endothelial growth factor (VEGF) signalling pathway (13 genes, of which 9 down-regulated; Fig. S3). The gene ontology classification shows corresponding gene changes grouped by their molecular function (Table 2). Among the statistically differentially expressed genes, changes were also found in the expression of genes involved in the pathways related to neuronal damage, Ca^2+^ regulation, gliosis, inflammation, synaptic transmission, and cytoskeletal structure (Tables 3 and 4). These changes were analyzed in more detail (as follows).

**Table 1:** KEGG-test for over-representation of the pathways in response to KA-treatment.

Entry number	Term or pathway	P Value	Odds Ratio	Count	Up-regulated	Down-regulated
rno00190	Oxidative phosphorylation	<0.01	3.99	26	7	19
rno03050	Proteasome	<0.01	7.36	12	6	6
rno04070	Phosphatidylinositol signaling system	<0.01	3.26	16	3	13
rno03010	Ribosome	<0.01	3.07	19	18	1
rno04720	Long-term potentiation	<0.01	2.99	18	4	14
rno00100	Biosynthesis of steroids	<0.01	5.31	8	3	5
rno00562	Inositol phosphate metabolism	<0.01	3.49	11	4	7
rno05010	Alzheimer's disease	0.01	4.21	9	5	4
rno03060	Protein export	0.01	8.77	4	0	4
rno04370	VEGF signaling pathway	0.02	2.22	13	4	9
rno04540	Gap junction	0.02	2.01	18	7	11
rno04912	GnRH signaling pathway	0.02	1.94	17	7	10
rno04730	Long-term depression	0.03	1.98	14	2	12
rno00260	Glycine, serine and threonine metabolism	0.03	2.71	8	4	4
rno00910	Nitrogen metabolism	0.05	3.75	4	0	4

**Table 2:** Classification of the gene ontology by the molecular function as revealed by the microarray study in juvenile rats after SE.

Entry number	Term	Number of altered genes
MF00042	Nucleic acid binding	406
MF00213	Non-receptor serine/threonine protein kinase	213
MF00262	Non-motor actin binding protein	126
MF00075	Ribosomal protein	94
MF00101	Guanyl-nucleotide exchange factor	92
MF00250	Serine protease inhibitor	81
MF00123	Oxidoreductase	67
MF00141	Hydrolase	65
MF00072	Translation initiation factor	65
MF00082	Transporter	57
MF00107	Kinase	56
MF00267	Membrane traffic protein	52
MF00137	Glycosyltransferase	47
MF00264	Microtubule family cytoskeletal protein	45
MF00097	G-protein	45
MF00211	Kinase activator	43
MF00170	Ligase	37
MF00108	Protein kinase	37
MF00283	Ubiquitin-protein ligase	36
MF00113	Phosphatase	35
MF00021	Neuropeptide	34
MF00255	Non-motor microtubule binding protein	34
MF00126	Dehydrogenase	33
MF00051	Helicase	27
MF00239	Phosphatase inhibitor	25
MF00094	Kinase modulator	25
MF00243	DNA helicase	25
MF00077	Chaperone	23
MF00270	Membrane traffic regulatory protein	22
MF00268	Vesicle coat protein	22
MF00236	Exoribonuclease	20
MF00218	Calmodulin related protein	19
MF00269	SNARE protein	14
MF00139	Acyltransferase	13
MF00121	Aminoacyl-tRNA synthetase	13
MF00110	Nucleotide kinase	13
MF00119	Synthase	12
MF00147	Deaminase	11
MF00116	Nucleotide phosphatase	11
MF00150	Glycosidase	11
MF00263	Other actin family cytoskeletal protein	9
MF00088	Apolipoprotein	9
MF00161	Decarboxylase	8
MF00155	Phosphorylase	5
MF00199	Storage protein	5
MF00200	Myelin protein	5
MF00115	Carbohydrate phosphatase	4
MF00007	Interferon receptor	3

**Table 3:** Differential expression of genes in the CA1 region of KA-treated rats compared to control rats: genes associated with neuronal damage, calcium-binding, gliosis and inflammation.

Gene databank accession no.	Gene name	Gene symbol	Fold change
	Genes associated with neuronal damage		
NM_053812	Bcl2-antagonist killer 1	Bak1	+3.31
NM_022698	Bcl2-associated death promoter	Bad	+1.10
NM_053586	Cytochrome c oxidase subunit 5b	Cox5b	+1.03
NM_182819	Cytochrome c oxidase subunit 7b	Cox7b	−1.07
NM_145783	Cytochrome c oxidase subunit 5a	Cox5a	−1.12
NM_017309	Protein phosphatase 3, regulatory subunit B, alpha isoform (calcineurin B, type I)	Ppp3r1	−1.94
	Genes associated with calcium-binding		
NM_013002	Purkinje cell protein 4	Pcp4	+13.3
XM_215607	S100 calcium binding protein A13	S100a13	+7.20
XM_579178	S100 calcium binding protein A1	S100a1	+5.10
XM_342291	S100 calcium binding protein A16	S100a16	+4.49
NM_013191	S100 calcium binding protein B	S100b	+3.34
NM_133605	Calcium/calmodulin-dependent protein kinase II gamma	Camk2g	+1.61
NM_021739	Calcium/calmodulin-dependent protein kinase II beta	Camk2b	+0.66
NM_017326	Calmodulin 2	Calm2	−1.05
NM_031662	Calcium/calmodulin-dependent protein kinase kinase 1, alpha	Camkk1	−1.06
XM_579543	Calmodulin 1	Calm1	−1.28
NM_017195	Growth associated protein 43	Gap43	−1.89
NM_031984	Calbindin 1	Calb1	−4.99
	Genes associated with gliosis and inflammation		
XM_579387	Glial fibrillary acid protein	Gfap	+13.7
NM_012823	Annexin A3	Anxa3	+7.84
NM_053960	Chemokine (C-C motif) receptor 5	Ccr5	+6.51
NM_017320	Cathepsin S	Ctss	+6.47
NM_013069	CD74 antigen (invariant polypeptide of major histocompatibility complex, class II antigen-associated)	Cd74	+6.10
NM_198740	Major histocompatibility complex, class II, DM beta	Hla-dmb	+5.84
NM_138828	Apolipoprotein E	Apoe	+3.76
NM_012512	Beta-2 microglobulin	B2m	+3.75
NM_012837	Cystatin C	Cst3	+3.58
NM_017232	Prostaglandin-endoperoxide synthase 2	Ptgs2	+2.48
NM_031560	Cathepsin K	Ctsk	−3.21
NM_019310	Interleukin 8 receptor, alpha	Il8ra	−6.08

#### Gene changes related to neuronal damage and calcium-binding proteins

Only a few genes involved in the apoptotic pathway were changed, i.e. BAD (+1.1 fold), Bcl2-antagonist/killer 1 (+3.31) (involved in cytochrome c release from the mitochondria), calcineurin B (−1.94), and specific components of the cytochrome c oxidase complex (see Tables 1 and 3; and Fig. S1). The most significantly influenced pathway was that for the oxidative phosphorylation (Fig. S1) implicating that the primary responses to the excitoxic damage after KA-induced SE involves oxidative mechanisms.

The expression of calmodulin 1 (CaM1) and calmodulin 2 (CaM2) were down-regulated (−1.28 and −1.05, respectively). The expression of two specific CaM kinases, CaMKIIg and CaMKIIb, was up-regulated (+1.61 and +0.66, respectively), and only calcium/calmodulin-dependent protein kinase kinase 1, alpha (CaMKK1) was down-regulated (−1.06). Moreover, the calbindin gene was down-regulated (−5), whereas the expression of several S100 calcium-binding proteins was increased. Among the small neuronal proteins that bind CaM, the PEP-19 gene was the second most up-regulated gene in our study (+13.3), whereas the growth associated protein 43 (GAP-43) gene was down-regulated (−1.89).

#### Gene changes associated with gliosis and inflammation

The structural astrocytic protein GFAP was the most dramatically up-regulated gene (+13.7) after SE (Table 3). The expression of the protease inhibitor cystatin C gene was pronouncedly up-regulated (+3.58) together with one of its substrates, cathepsin S (+6.47). Also the annexin A3 (Anxa3) gene, which plays a role in the regulation of cellular growth, showed an increased transcriptome expression (+7.84).

The immunologically-related beta-2 microglobulin (+3.75) and HLA-DMB (+5.84) genes were highly up-regulated, as well as the chemokine receptor CCR5 (+6.51) and the glycoprotein CD74 (+6.1) genes. The interleukin 8 receptor (CXCR1) was highly down-regulated (−6.08), but prostaglandin synthase 2 (PTGS2), also known as cyclooxygenase-2 (COX-2), was up-regulated (+2.48). This inflammatory mediator is also associated to the VEGF signalling pathway, and we found activation of several genes in this pathway after SE (Table 1; Fig. S3). Apo E, another protein expressed in astrocytes, showed an increased expression (+3.76).

#### Gene changes associated with synaptic transmission

The γ-aminobutyric acid type A (GABA_A_) receptor α5 subunit gene expression was down-regulated (−2.71), whereas the expression of γ2 (+1.92) and β1 (+0.99) subunits were up-regulated (Table 4). We also detected a down-regulation of the gephyrin (−2.13) gene, and an up-regulation of the GABA_A_ receptor-associated protein (GABARAP; +1.81) gene. The gene expression of the N-methyl-D-aspartate (NMDA) receptor subunit NR2C was up-regulated (+3.78), while the NR2A subunit was down-regulated (−1.44) after SE. Furthermore, the α-amino-3-hydroxy-5-methyl-4-isoxazolepropionic acid (AMPA) receptor subunit GluR2 was down-regulated (−1.7). The concerted action of AMPARs and NMDARs in hippocampal CA1 pyramidal cells in the LTP pathway is shown in Fig. 4. The G protein-coupled receptor CB1 showed an up-regulated expression (+3.76).

**Table 4:** Differential expression of genes in the CA1 region of KA-treated rats compared to control rats: genes associated with synaptic transmission, voltage-gated channels, ribosomes and cytoskeleton.

Gene databank accession no.	Gene name	Gene symbol	Fold change
	Genes associated with synaptic transmission		
NM_012575	Glutamate receptor, ionotropic, NMDA 2C	Grin2c	+3.78
NM_012784	Cannabinoid receptor 1	Cnr1	+3.76
NM_031853	Diazepam binding inhibitor	Dbi	+2.37
NM_183327	GABA-A receptor, subunit gamma 2	Gabrg2	+1.92
NM_172036	GABA-A receptor associated protein	Gabarap	+1.81
NM_012956	GABA-A receptor, subunit beta 1	Gabrb1	+0.99
NM_153308	Glutamate receptor, ionotropic, NMDA-associated protein 1 (glutamate binding)	Grina	+0.87
XM_579337	Glutamate receptor, ionotropic, NMDA 2A	Grin2a	−1.44
NM_017261	Glutamate receptor, ionotropic, AMPA 2	Gria2	−1.7
XM_579467	Gephyrin	Gphn	−2.13
NM_017295	GABA-A receptor, subunit alpha 5	Gabra5	−2.71
	Genes associated with voltage-gated channels		
NM_021688	Potassium channel, subfamily K, member 1	Kcnk1	+3.51
NM_172042	Potassium channel, subfamily K, member 2	Kcnk2	+3.32
NM_012856	Potassium voltage gated channel, Shaw-related subfamily, member 1	Kcnc1	+1.66
NM_139216	Potassium voltage gated channel, Shaw-related subfamily, member 2	Kcnc2	+1.62
NM_053351	Calcium channel, voltage-dependent, gamma subunit 2	Cacng2	+1.19
NM_139097	Sodium channel, voltage-gated, type III, beta	Scn3b	−1.14
NM_013186	Potassium voltage gated channel, Shab-related subfamily, member 1	Kcnb1	−1.51
NM_012647	Sodium channel, voltage-gated, type 2, alpha 1 polypeptide	Scn2a1	−1.58
NM_024139	Calcium binding protein p22	Chp	−1.64
XM_217464	Calcium binding protein 39	Cab39	−1.91
	Genes associated with ribosomes and cytoskeleton		
NM_017138	Laminin receptor 1 (protein SA)	Rpsa	+3.75
NM_022504	Ribosomal protein L36	Rpl36	+3.30
NM_053597	Ribosomal protein S27	Rps27	+2.75
NM_031110	Ribosomal protein S11	Rps11	+2.39
NM_031102	Ribosomal protein L18	Rpl18	+1.95
NM_017150	Ribosomal protein L29	Rpl29	+1.93
NM_022949	Ribosomal protein L14	Rpl14	+1.61
NM_013226	Ribosomal protein L32	Rpl32	+1.41
NM_031065	Ribosomal protein L10A	Rpl10a	+1.34
NM_017151	Ribosomal protein S15	Rps15	+1.31
XM_575183	Ribosomal protein L21	Rpl21	+1.21
NM_031109	Ribosomal protein S10	Rps10	+1.18
NM_013066	Microtubule-associated protein 2	Mtap2	+1.12
XM_579666	Tubulin, beta 3	Tubb3	−1.08
NM_031783	Neurofilament, light polypeptide	Nefl	−1.62

#### Validation of selected genes of interest by immunohistochemistry

**Figure 5:**
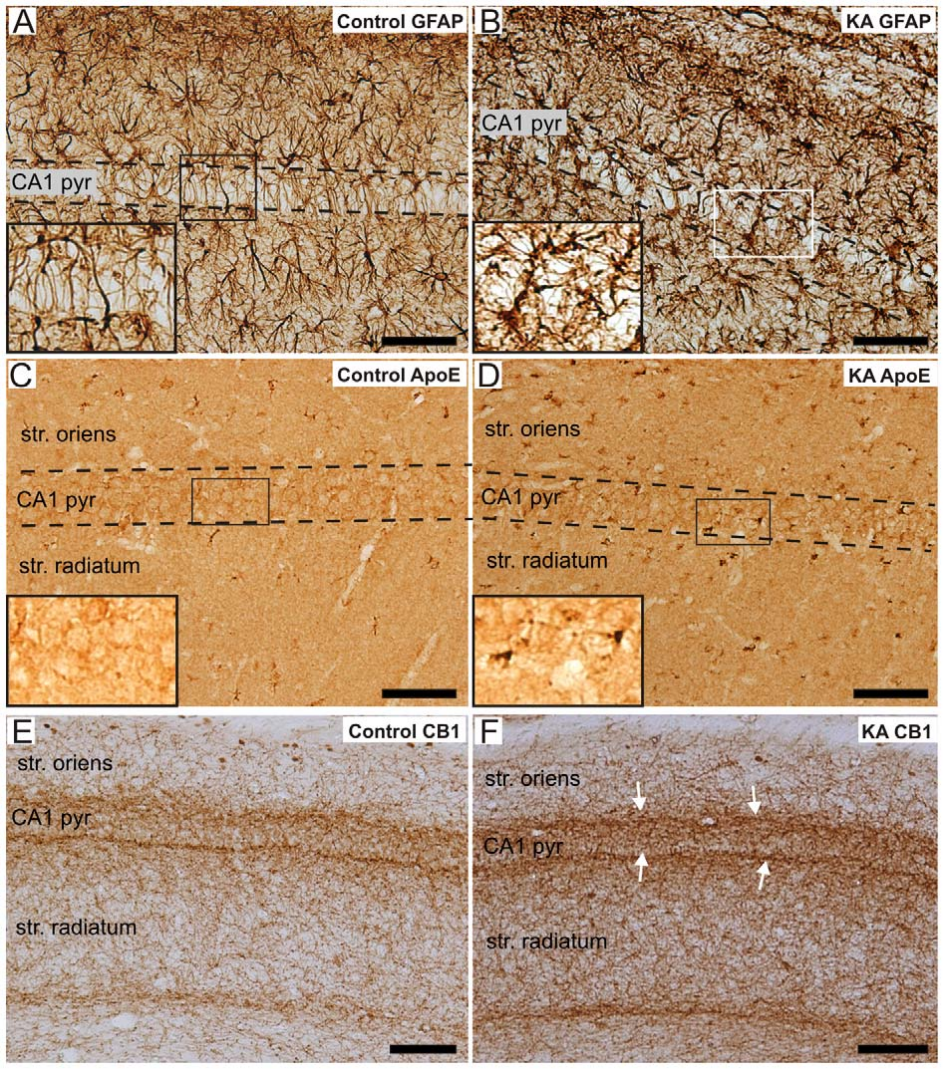
Validation of the microarray results by immunohistochemical staining in control and KA-treated rats. Immunoreactivity of GFAP in a representative control (A) and KA-treated rat (B). Note the increase in GFAP immunoreactivity and the morphological change in astrocytes of the KA-treated rat (insets in A and B). Figs 5 C and D show the immunoreactivity of apo E in a control and KA-treated rat, respectively. Note the increase in apo E immunostaining in some cells within the CA1 pyramidal cell layer of the KA-treated rat compared to the control rat (insets in C and D, respectively). The CB1 immunoreactivity was enhanced more pronouncedly in a KA-treated rat (F) than in a control rat (E) in the borders of the CA1 pyramidal layer with both the stratum oriens and radiatum (white arrows in F). Scale bars: 75 µm in A–D, and 100 µm in E-F. Abbreviations: apo E, apolipoprotein E; CB1, cannabinoid type 1 receptor; CA1 pyr, stratum pyramidale of CA1; GFAP, glial fibrillary protein; KA, kainic acid; str., stratum.

The validation of the microarray results was carried out by immunohistochemical detection of the selected genes of interest, i.e. GFAP, apo E, CB1, PEP-19 and CXCR1. Figs. 5A and 5B show representative GFAP immunostainings of the CA1 region in a control and KA-treated rat, respectively. GFAP was heavily increased in astrocytes of the KA-treated rats (see Fig. 5B and the inset). Immunopositive apo E cells appeared in the CA1 pyramidal cell layer of KA-treated rats (Fig. 5D) compared to the control rats, in which no positively labelled cells were detected (Fig. 5C). Furthermore, the enhanced apo E immunoreactivity was also detected in the stratum oriens and radiatum of the CA1 region. One of the pronouncedly increased genes after SE in the transcriptome analysis was the CB1 gene, which was specifically enhanced in the borders of the CA1 pyramidal cell layer with both the stratum oriens and radiatum. Figs. 5E and F show the CB1 immunostaining in the CA1 region of a control and KA-treated rat, respectively.

**Figure 6:**
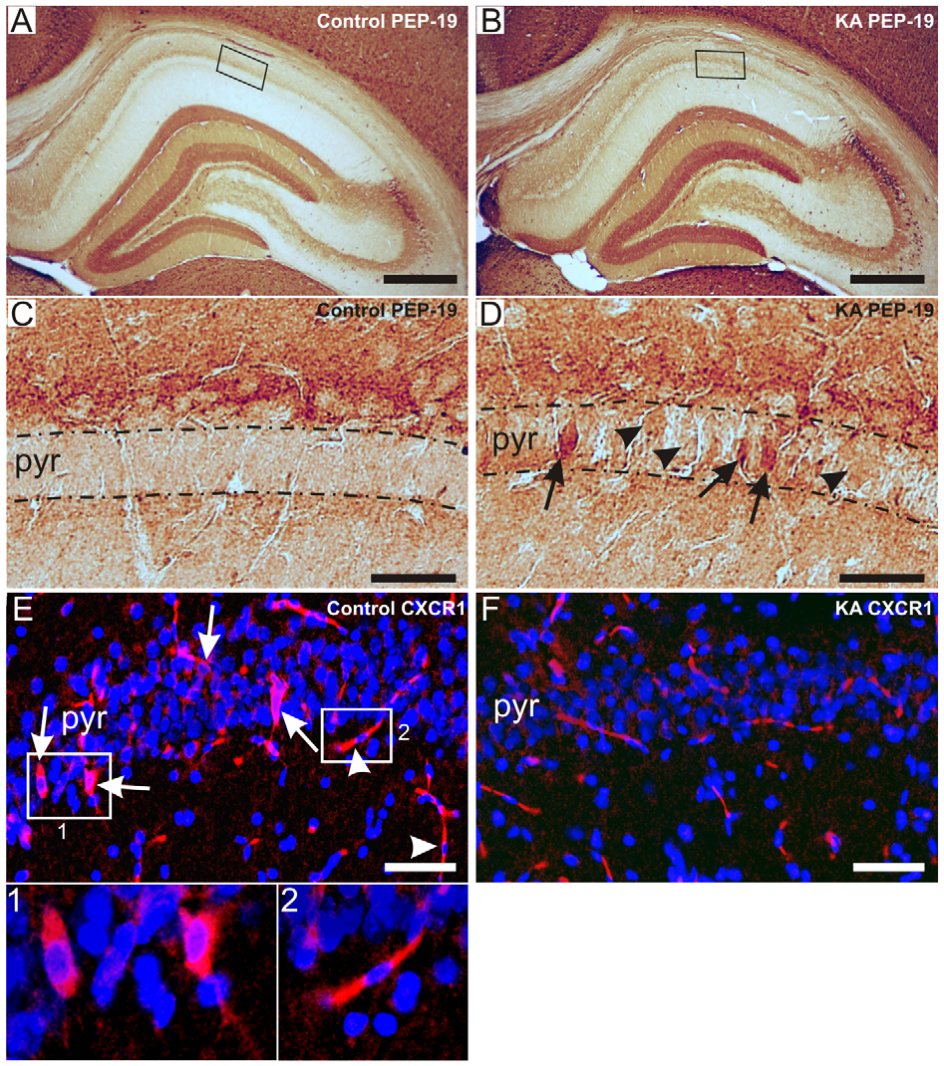
Validation of the microarray results by immunohistochemical staining of PEP-19 and CXCR1 in control and KA-treated rats. Immunoreactivity of PEP-19 in the whole hippocampus in a representative control (A) and KA-treated rat (B). Figs. 6C and Dshow the CA1 region of A and B, respectively, at the higher magnification. Note the lack of PEP-19 immunoreactivity in the CA1 pyramidal layer in the control rat (C), while the staining was weak in the CA1 layer (D, arrowheads), and greatly enhanced in single neurons (D, arrows) in the KA-treated rat. Figs. 6E and Fshow the CXCR1 immunoreactivity in the CA1 region of a control and KA-treated rat. In control rats, immunoreactivity occurred in discrete neurons in the CA1 pyramidal layer (E, arrows and insert 1), and in vascular endothelial cells (E, arrowheads and insert 2). In KA-treated rats, no immunoreactive neurons could be detected (F). Scale bars: 200 µm in A–B, 40 µm in C–D, and 75 µm in E-F. Abbreviations: CXCR1, interleukin 8 receptor; PEP-19, Purkinje cell protein 4; pyr, stratum pyramidale.

PEP-19 and CXCR1 were among the most up-regulated and down-regulated genes, respectively. Figs. 6A and 6B show representative PEP-19 immunostainings of the whole hippocampus in a control and KA-treated rat, respectively. With higher magnification, highly enhanced PEP-19 immunoreactivity was observed in some CA1 neurons of KA-treated rats (Fig. 6D, arrows), whereas in the majority of neurons the staining was weak (Fig. 6D, arrowheads). Meanwhile, no PEP-19 immunoreactive neurons could be detected in the CA1 pyramidal layer in control rats (Fig. 6C). Figs. 6E and 6F show representative CXCR1 fluorescent immunostainings of the CA1 region in a control and KA-treated rat, respectively. Positive CXCR1 immunostaining was detected in discrete CA1 neurons of control rats (6E, arrows and insert 1), as well as in vascular endothelial cells as identified by their location and shape (arrowheads and insert 2). In KA-treated rats, no clearly CXCR1 immunoreactive neurons were observed in the CA1 layer (Fig. 6F). The immunostaining results are in line with our corresponding gene expression findings as verified by the microarrays.

## Discussion

In this study we elucidated the consequences of KA-induced SE on gene expression in the CA1 pyramidal cell region at the sub-acute phase of epileptogenesis, i.e. one week after SE, in P21 rats, the age roughly comparable to a juvenile child (reviewed in [27]). The CA1 region was microdissected by laser, which gave us an opportunity to focus specifically on this region selectively damaged by SE at this age [7], [23]. It is, so far, incompletely known when the epileptogenic process begins, and which molecular changes are relevant to it. Earlier results, however, suggest that neurodegeneration, that occurs in the hilus and CA1 pyramidal cell layer during the early phase of epileptogenesis, could be one reliable indicator of the epileptogenic process at least in adult rats (reviewed in [28]). A total of 1592 genes were differently expressed in the CA1 sub-region of the KA-treated compared to their age-matched control rats. These genes were involved in several pathways regulating e.g. neuronal damage, Ca^2+^ homeostasis, glial reactivity, inflammation, and synaptic transmission. The observed alterations in gene expression of the pathways of interest are discussed in the light of earlier studies in adult rats, and their postulated significance in epileptogenesis in juvenile rats.

### Differential expression of genes related to neuronal damage and neuroprotection

#### The mechanism of SE-induced CA1 pyramidal cell damage and the role of calcium

In our model, the CA1 neurons were selectively damaged in the P21 rats one week after SE. However, our results suggest that the activation of the apoptotic pathway may not be involved in the damage process. In adult rats both apoptotic and necrotic mechanisms contribute to neuronal degeneration in all hippocampal subregions after KA-induced SE [29], [30], followed by progressive neuronal loss, detectable within one week after SE [5], [31], [32]. SE-induced up-regulation of genes associated with apoptosis, such as caspase-3, p53, and BAX, have also been detected in adult rats [13], [33], [34], [35]. At variance in the P21 rats, the expression of these genes did not significantly differ from those of the control rats, although the positive FJB-staining of CA1 neurons persisted at least for one week. The contribution of non-apoptotic mechanisms in the CA1 region after SE is also corroborated by our recent finding that the expression of Bax and caspase-3 proteins remained unchanged after SE in P21 rats [23].

The S100 proteins are a family of calcium-binding, EF-hand proteins localized to astrocytes. They are involved in Ca^2+^ regulation in a variety of intracellular pathways [36]. The up-regulation of the S100B protein has been found in previous studies [37], [38] suggesting an activation of pathways, which lead to increased intracellular Ca^2+^ concentrations in astrocytes [36]. The calbindin gene, abundantly expressed in dentate gyrus (DG) granule cells, and to some extent also in CA1 pyramidal neurons [22], [39], was now down-regulated. This is in accordance with earlier studies in adult rats, in which the calbindin gene has been down-regulated in the hippocampal subregions 1–4 weeks after pilocarpine-induced SE [40], [41], and its immunoreactivity attenuated in CA1 pyramidal neurons one week after KA- or electrically-induced SE [42], [43], suggesting for an attenuated regulation of intracellular Ca^2+^ levels upon stimulation [39], [41]. Another highly up-regulated transcript in our study was that of PEP-19, a small neuronal protein involved in the calcium-binding to CaM [44]. PEP-19 is highly expressed in hippocampal CA2 pyramidal cells, which display resistance to epileptic damage possibly due to their higher Ca^2+^ buffering capacity [45]. Furthermore, PEP-19 has shown neuroprotective properties in cell cultures, these possibly being mediated through CaM inhibition that could lead to enhanced resistance of neurons to calcium-mediated toxicity [46], [47], which makes it tempting to speculate that this protein is neuroprotective also in our SE model. The reduced CaM expression in our study is also interesting since there is extensive evidence that CaM plays a critical role in the induction of cell death following Ca^2+^ overload, and inhibition of CaM function can protect neurons from death [48], [49]. Overall, our present results suggest that genes in the pathways regulating the cellular Ca^2+^ homeostasis are one of the major targets modified by SE in juvenile rats. Also recent studies in adult rats suggest that those neurons that survive after seizures exhibit prolonged alterations in neuronal Ca^2+^ dynamics that could play an important role in the induction and maintenance of prolonged plastic changes characteristic for the epileptic phenotype [41], [50].

### Genes associated with gliosis and inflammation

The structural astrocytic protein GFAP was the most dramatically up-regulated gene after SE in keeping with earlier studies in adult experimental epilepsy models, i.e. one day after KA-induced SE [14], 14 days after pilocarpine-induced SE [21], and 8 days after electrically-induced SE [51]. In line with the gene expression results, enhanced GFAP protein expression has been detected within days after SE in the hippocampus of various experimental epilepsy models in the immature (P15–P21) [7], [52], and adult rats [38], [53], [54]. Also the morphology of astrocytes is altered by SE, fibrous astrocytes with long processes being replaced by reactive astrocytes with thickened, heavily GFAP-postitive processes [7], [37]. Comparable changes were also detected in our study corroborating the significant role of glial cells at the early phase of epileptogenesis. The increased GFAP expression is consistent with earlier studies of glial cell activation known to accompany different types of neural injury [55], [56].

The expression of the protease inhibitor cystatin C gene was likewise pronouncedly up-regulated in keeping with earlier studies, in which it has been up-regulated in reactive astrocytes, activated microglia, and in CA1 pyramidal neurons after electrically-induced SE in adult rats [43], [51]. Functionally, cystatin C may protect cells as it inhibits the proteolytic effect of cathepsins [57], [58]. Interestingly, also the gene expression of the lysosomal cysteine protease cathepsin S has been up-regulated in the hippocampus after SE in adult rats [17], as well as in microglial cells surrounding the degenerating CA1 pyramidal neurons in juvenile mice [59]. Although the members of the cathepsin family may contribute to neuronal apoptosis when secreted by activated microglia [60], [61], the significance of cathepsin S gene up-regulation observed now remains speculative, since our other data suggest that apoptosis seems not to be activated by SE in P21 rats.

Several genes mediating inflammatory processes were also highly up-regulated, such as the chemokine receptor CCR5, and the glycoprotein CD74. This is in line with earlier studies which have shown that seizures induce the expression of different chemokines and cytokines in adult and immature brain [7], [16], [52]. CCR5 is expressed in both neuronal and glial cells in the rat brain. Moreover, its expression is time-dependently enhanced in the adult rat hippocampus after KA-induced seizures concomitant to neurodegeneration [62]. It has been proposed that the CCR5-expressing glial cells could play a distinct pathophysiological role in contributing to the progression of injury and/or be a part of intrinsic repair mechanisms [62]. The CXCR1 gene, expressed both in hippocampal neurons and astrocytes [63], as well as in endothelial cells [64], [65], was now down-regulated. CXCR1 is a G-protein-coupled receptor activated by the pro-inflammatory chemokine, interleukin-8 (IL-8 or CXCL8), that is up-regulated under acute inflammatory conditions, e.g. after ischemia in the adult rabbit brain [66]. IL-8 may reduce Ca^2+^ currents through CXCR1, which may modify neuronal excitability [67]. Indeed, a neutralizing anti-IL-8 antibody has significantly reduced ischemia-induced brain damage [66], further suggesting that inhibition of IL-8 binding to its receptor CXCR1 is another potential target for inhibiting the IL-8 effects [68], and the decreased CXCR1 expression may play a neuroprotective role. Moreover, activation of the COX-2 gene occurred in our model in accordance with earlier transcriptome studies in adult rats [13], [16], [69], and its enhanced immunoreactivity has recently been detected in several hippocampal subregions, including the CA1 region after SE [23]. This inflammatory mediator is also associated to the VEGF signalling pathway supporting the idea of recent studies that seizures promote angiogenesis [13], [70], which could contribute to the recovery process after SE.

Apo E is postulated to modulate the astroglial response to damage, and to play a role in neuronal repair and re-innervation of the damaged circuitry [71]. This molecule is the major constituent of brain lipoproteins abundantly expressed in astrocytes [72]. It was now highly up-regulated in accordance with earlier studies in the adult rat hippocampus after systemic KA- and electrically-induced SE [51], [73]. Taken together, our current transcriptome results together with the earlier studies by other groups strongly favour the idea that activated microglia and astrocytes play a pivotal role in immune responses as well as in the damage and repair processes of the CA1 pyramidal neurons in juvenile P21 rats after SE.

### Genes associated with neurotransmission and signal transduction

#### GABA_A_, NMDA and cannabinoid receptor signalling

There is extensive evidence that seizures trigger alterations in the GABAergic inhibition as well as in the GABA_A_ receptor subunit expression in the adult [74], [75], [76] and immature (P9) rat brain [77]. Corroborating our findings, the observed down-regulation of the α5 subunit and up-regulation of the γ2 subunit along with a decreased δ and increased α4 subunit expression, have been found in several adult experimental TLE models [16], [69], [74], [78], [79]. Interestingly, changes in these subunits could alter both tonic and phasic inhibition, as the δ, α4 and α5 subunits are primarily found in extrasynaptic GABA_A_ receptors, which mediate tonic inhibitory currents [80], [81], [82], whereas the γ2 subunit is mainly localized to synaptic receptors that play a major role in phasic inhibition [83], [84]. A decreased α5 subunit expression has, however, resulted in enhanced tonic inhibition in CA1 pyramidal cells in adult rats after pilocarpine-induced seizures [85] supporting the idea that compensatory up-regulation of other extrasynaptic GABA_A_ receptors, possibly those containing α4 subunit, may take place. Thus, when the α5 or δ subunit expression is decreased, the γ2 subunit is translocated from synaptic to perisynaptic sites and increases its partnership with the α4 subunit in the epileptic brain [78].

We also detected a down-regulation of the gephyrin gene and an up-regulation of the GABARAP gene in accordance with the results obtained from the hippocampi of humans with TLE [86]. Gephyrin and GABARAP are essential proteins in the postsynaptic clustering of γ2 subunit-containing GABA_A_ receptors [87], [88], and the gephyrin down-regulation could further corroborate the hypothesis that the γ2 subunit translocates from synaptic to perisynaptic sites during epileptogenesis.

In hippocampal CA1 pyramidal cells, activation of NMDA receptors and subsequent Ca^2+^ influx is connected to the induction of synaptic plasticity through LTP [89]. The low NR2A and high NR2C transcriptome expression detected now implicates a recapitulation of an immature NMDA receptor phenotype after SE, as the NR2B and NR2C subunits are highly expressed in the developing hippocampus, and around the third postnatal weeks these two subunits are replaced by NR2A and NR2D [90]. Changes in NMDA receptor subunits have been proposed to cause deficits in long-term spatial learning ability [91]. Furthermore, the increased NR2C subunit expression in our model may also be associated with the induction of CaM kinase II isoforms, which are involved in the NMDA receptor-mediated Ca^2+^ signalling in an *in vitro* model of epilepsy [92].

Decreased expression of the AMPA receptor GluR2 subunit gene has earlier been detected in the CA3 region shortly after KA-induced and electrically-induced SE in adult rats, in keeping with our present finding [16], [93]. During the first two postnatal weeks in rats, the expression of the GluR2 subunit-containing AMPARs are increased in the CA1 region [90], leading to attenuated Ca^2+^ influx, since this subunit exhibits low permeability to Ca^2+^ [94]. The reduced expression of the GluR2 subunit in the juvenile rats after SE could therefore lead to Ca^2+^ overload in CA1 pyramidal neurons, and contribute to their damage.

The G-protein-coupled CB1s are mainly found on the nerve terminals of cholecystokinin-containing GABAergic interneurons in the hippocampus [95]. Exogenous cannabinoids act on presynaptic CB1s and block voltage-gated Ca^2+^ channels and reduce Ca^2+^ influx necessary for GABA release [95], [96]. This can result in decreased GABA-mediated inhibitory currents [97] that could facilitate LTP induction [98]. The up-regulated CB1 receptor gene expression in our study is in line with earlier studies, in which CB1 receptors were up-regulated after seizures in adult rats [99], [100]. This suggests that the cannabinoid system is one regulator of seizure activity. Moreover, KA-induced seizures increase endocannabinoid levels in the normal mice hippocampus, and induce more severe neuronal damage in the hippocampus of CB1 knockout mice [101]. It is thus possible that also in our juvenile rats SE-induced CB1 activation may trigger endocannabinoid release that could serve as a neuroprotective factor.

### Concluding remarks

The transcriptome analysis of the hippocampal CA1 subregion one week after SE in P21 rats with respect to their age-matched control rats resulted in differentially expressed genes associated to several pathways. Induced expression of several genes related to the Ca^2+^ signalling pathway, and the glial and inflammatory responses were observed, but only a few genes that were related to the apoptotic pathway. The observed absence of apoptosis may be associated with the developmental stage of the CA1 pyramidal neurons in P21 rats and/or a possible beneficial role of astrocytic cells in the repair processes of pyramidal neurons. The vast majority of the CA1 pyramidal neurons, although damaged (as indicated by positive FJB-staining), survived at least for one week after SE, but the altered gene expression suggest on-going epileptogenic processes in this sub-region. The alterations in the expression of several GABA_A_ receptor subunits and that of CB1 may, on the other hand, be of importance for seizure regulation. Moreover, several molecular mechanisms occurring during epileptogenesis in our juvenile rats are similar to those in adult rats. However, the highly up-regulated expression of the calcium-binding protein PEP-19 gene, and the down-regulation of the CXCR1 gene, involved in inflammation, seem to be specific for this age. Further studies are needed to reveal whether these molecules have a possible role in neuroprotection, and whether they can provide new leads to better understand the mechanisms of epileptogenesis in the juvenile hippocampus.

## Supporting Information

Figure S1 The KEGG-derived oxidative phosphorylation pathway. Oxidative phosphorylation was the most significantly influenced pathway in our transcriptome analysis. Specific components of the cytochrome c oxidase complex were also activated, and these genes are encircled in the figure. The table below shows the entire list of the up- or down-regulated genes on the microarray related to this pathway.
(0.99 MB TIF)

Figure S2 The KEGG-derived ribosomal pathway. The figure shows gene changes found in the various components of the ribosomal machinery, with the genes altered on the microarray encircled. An increased expression of many genes encoding ribosomal proteins was found after SE as shown in the table. This could indicate that the cells actively change their protein synthesis capacity after seizures.
(0.59 MB TIF)

Figure S3 The KEGG-derived VEGF signaling pathway. The VEGF signalling pathway with the genes activated on the microarray encircled. In this pathway, 13 genes were changed, of which 9 were down-regulated as seen in the table.
(0.38 MB TIF)
