# Supplementary figures and images for: Transcriptome Analysis of the Hippocampal CA1 Pyramidal Cell Region after Kainic Acid-Induced Status Epilepticus in Juvenile Rats
Source: PLoS One. 2010 May 20;5(5):e10733. doi: 10.1371/journal.pone.0010733 (PMC2873964; doi:10.1371/journal.pone.0010733)

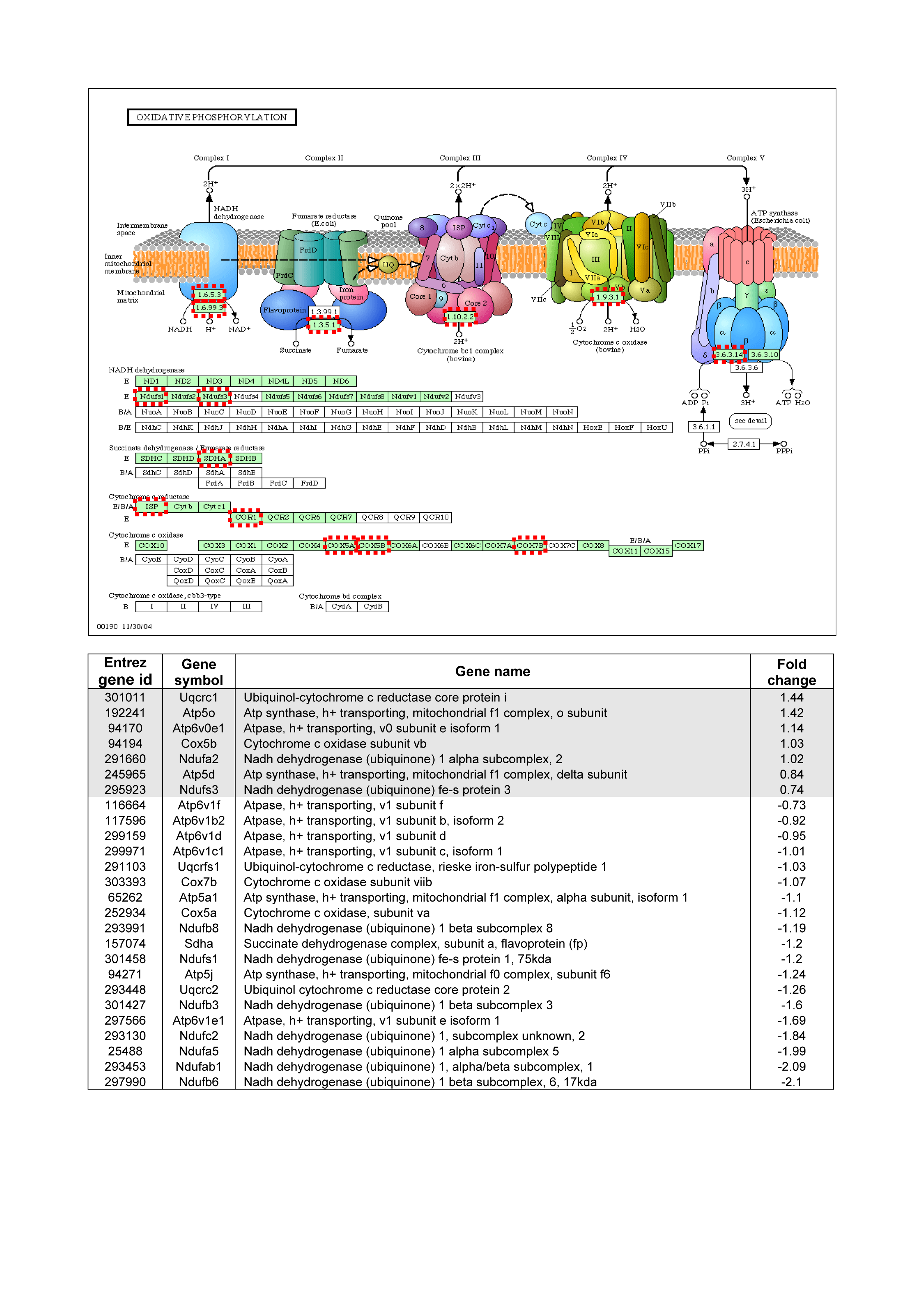

Supplement: Figure S1 — The KEGG-derived oxidative phosphorylation pathway. Oxidative phosphorylation was the most significantly influenced pathway in our transcriptome analysis. Specific components of the cytochrome c oxidase complex were also activated, and these genes are encircled in the figure. The table below shows the entire list of the up- or down-regulated genes on the microarray related to this pathway. (0.99 MB TIF) [file pone.0010733.s001.tif]

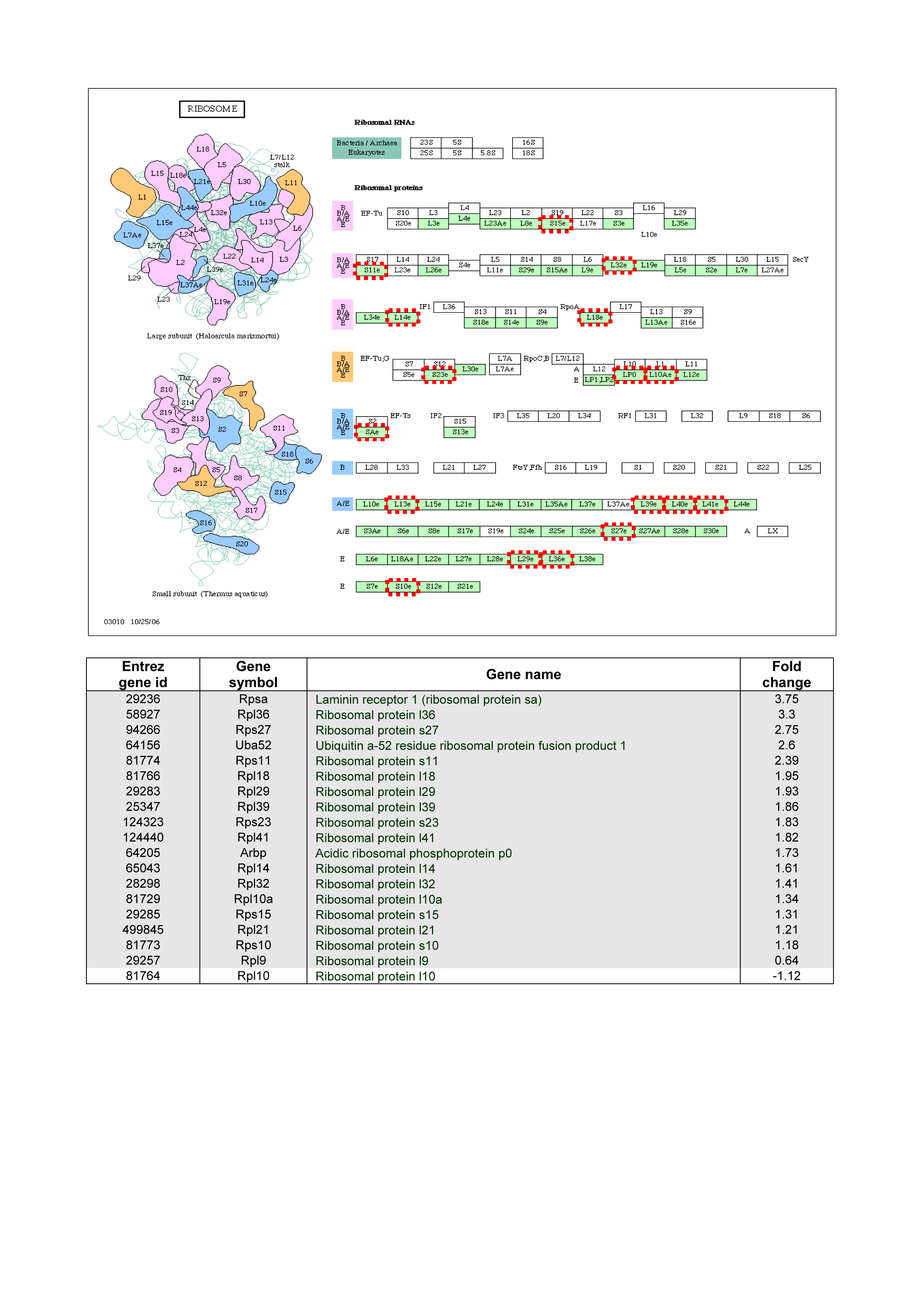

Supplement: Figure S2 — The KEGG-derived ribosomal pathway. The figure shows gene changes found in the various components of the ribosomal machinery, with the genes altered on the microarray encircled. An increased expression of many genes encoding ribosomal proteins was found after SE as shown in the table. This could indicate that the cells actively change their protein synthesis capacity after seizures. (0.59 MB TIF) [file pone.0010733.s002.tif]

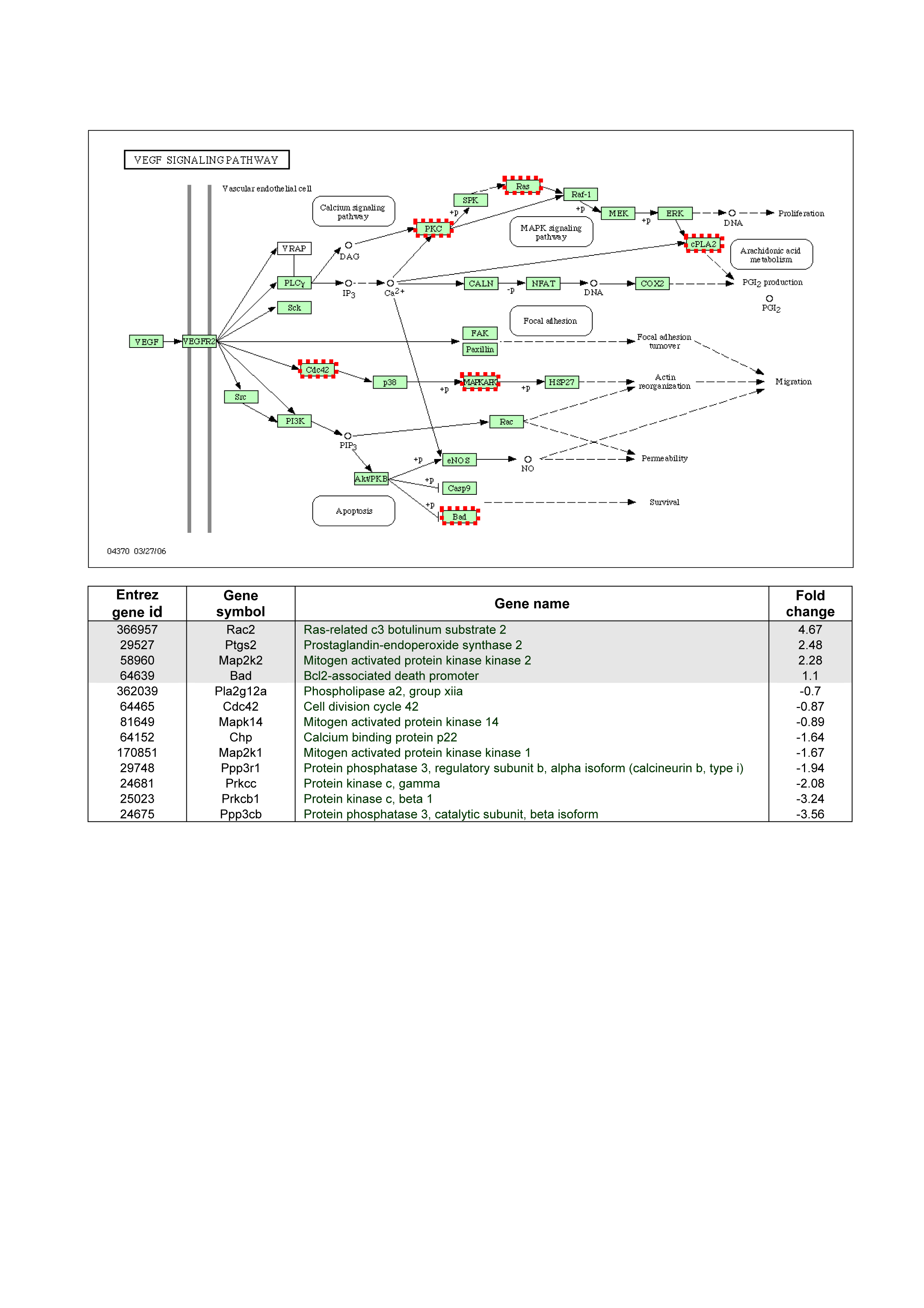

Supplement: Figure S3 — The KEGG-derived VEGF signaling pathway. The VEGF signalling pathway with the genes activated on the microarray encircled. In this pathway, 13 genes were changed, of which 9 were down-regulated as seen in the table. (0.38 MB TIF) [file pone.0010733.s003.tif]
